# Human adipose mesenchymal stem cell-derived exosomes alleviate fibrosis by restraining ferroptosis in keloids

**DOI:** 10.3389/fphar.2024.1431846

**Published:** 2024-08-16

**Authors:** Yuan Tian, Meijia Li, Rong Cheng, Xinyue Chen, Zhishan Xu, Jian Yuan, Zhiyong Diao, Lijun Hao

**Affiliations:** Plastic Surgery, Harbin Medical University, Harbin, China

**Keywords:** adipose-derived mesenchymal stem cells, fibrosis, ferritic, extracellular vesicles, GPx4

## Abstract

**Background:**

Keloid is a fibroproliferative disease with unsatisfactory therapeutic effects and a high recurrence rate. exosomes produced by adipose-derived mesenchymal stem cells (ADSC-Exos) have attracted significant interest due to their ability to treat fibrosis. However, the molecular mechanisms of ADSC-Exos in keloids remain inconclusive.

**Objective:**

Our study revealed the relationship between ferroptosis and fibrosis in keloids. Subsequently, this study aimed to explore further the anti-fibrotic effect of ADSC-Exos on keloids through ferroptosis and the potential underlying mechanisms.

**Methods:**

To investigate the impact of ferroptosis on keloid fibrosis, Erastin and ferrostatin-1 (fer-1) were utilized to treat keloid fibroblast. Keloid keloids treated with Erastin and fer-1 were cocultured with ADSC-Exos to validate the impact of ferroptosis on the effect of ADSC-Exos on keloid anti-ferrotic protein, peroxidase 4 (GPX4) and anti-fibrotic effects *in vivo* and *in vitro* by Western blot, as well as variations in iron metabolite expression, malondialdehyde (MDA), liposomal peroxidation (LPO) and glutathione (GSH) were analyzed. The effect of solute carrier family 7-member 11 (SLC7A11) silencing on ADSC-Exo-treated keloid fibroblast was investigated.

**Results:**

Iron metabolite dysregulation was validated in keloids. Fibrosis progression is enhanced by Erastin-induced ferroptosis. The anti-fibrotic effects of ADSC-Exos and fer-1 are related to their ability to prevent iron metabolism. ADSC-Exos effectively suppressed keloid fibrosis progression and increased GSH and GPX4 gene expression. Additionally, the use of Erastin limits the effect of ADSC-Exos in keloids. Furthermore, the effect of ADSC-Exos on keloids was associated with SLC7A11-GPX4 signaling pathway.

**Conclusion:**

We demonstrated a new potential mechanism by which anti-ferroptosis inhibits the progression of keloid fibrosis and identified an ADSC-Exo-based keloid therapeutic strategy. Resisting the occurrence of ferroptosis and the existence of the SLC7A11-GPX4 signaling pathway might serve as a target for ADSC-Exos.

## 1 Introduction

Keloids can lead to physical discomfort, functional difficulties, and aesthetically pleasing problems, all of which can trigger psychological discontent (Jeschke et al., 2023). The traditional treatment methods for keloids mainly include ionizing beams, hormone injection, and cryosurgery. However, effective treatment methods are needed to guarantee the recurrence rate after treatment (Kadunc and Brunner, 2024). Therefore, identifying a treatment method that can target the pathogenesis of keloids is the key to solving this problem.

Ferroptosis is a recently discovered process that regulates cell necrosis (Jiang et al., 2021). It has been scientifically linked to several diseases. Changes in iron homeostasis, for example, have been associated with an increased probability of end-stage renal disease (ESKD) (Yu et al., 2020; Cai et al., 2023), atherosclerotic cardiovascular disease (Fang et al., 2023), and diabetes (Hoy et al., 2021). Preventing ferroptosis can substantially reduce the number of myofibroblast-like cells, which leads to less fibrosis. Ferroptosis controls fibroblast apoptosis and fibrosis in a complex and tissue-specific manner (Du et al., 2023). The role of ferroptosis in keloids is currently unclear. In our previous study, we compared the expression of ferroptosis genes in keloid fibroblast (KF) and normal fibroblast. The results showed that keloid fibrosis was associated with ferroptosis.

MSC-derived extracellular vesicles are innovative cell-free therapeutics for immunomodulation and regenerative purposes (Xiao et al., 2021). Human adipose-derived stem cells (ADSCs) are a vital source of stem cells because of their simplicity of utilization, self-renewal ability, minimum immunogenicity, high proliferation rate, and capacity to undergo differentiation into different lineages (Hoang et al., 2022). Exosomes, one of the most common types of extracellular vesicles, function in intercellular communication (Baumann, 2021). Some investigators believe that exosomes from human adipose-derived mesenchymal stem cells (ADSC-Exos) potentially restrict excessive collagen formation in fibroblasts. By activating the PI3K/AKT/mTOR signaling pathway, ADSC-Exos inhibited the expression of profibrogenic proteins and epithelial-to-mesenchymal transition (EMT). (Zhang et al., 2023). Furthermore, certain investigators have achieved unique medicinal properties. Excessive scar formation can be remediated by employing ADSC-Exos as transport carriers for pharmaceuticals and noncoding RNAs (Zhu et al., 2020; Li et al., 2021; Yuan et al., 2021). Consequently, it is crucial to elucidate the potential mechanism underlying the inhibitory effect of ADSC-Exos on the progression of keloid fibrosis.

ADSC-Exos inhibits ferroptosis induced by excessive inflammation and upregulates the expression of glutathione peroxidase 4 (GPX4) in human brain microvascular endothelial cells (Wu et al., 2024). ADSC-Exos can effectively improve the neurobehavior of mice and improve ferroptosis-related outcomes (Wang Y. et al., 2023). In this study, we identified an innovative approach in which ADSC-Exos inhibited the myofibroblast differentiation of KF by decreasing ferroptosis in keloids.

## 2 Materials and methods

### 2.1 Tissue and cell sources

The First Affiliated Hospital of Harbin Medical University’s Ethics Committee approved the collection of human tissue samples, and the study was conducted in accordance with the 2013 Declaration of Helsinki (No. 2023IIT115). For every tissue biopsy, informed consent was obtained. Samples from mature keloids and adipocytes were collected from plastic surgery patients. Professional dermatologists and plastic surgeons test the clinical nature of keloids.

ADSCs were isolated from subcutaneous adipose tissue of patients who underwent lipoplastic surgery and were freely available. Human keloid and standard skin fibroblast samples were obtained from patients who underwent surgery to eliminate a keloid and its surrounding normal skin or from the same patient’s normal skin from the donor location of skin graft surgery; 16 different patients were included in this study. Under low glucose conditions, Dulbecco’s modified Eagle’s medium (DMEM, Gibco, United States), keloid fibroblast (KF), normal skin fibroblasts, and ADSCs were cultivated. The medium also included 100 IU/mL penicillin, 10 mg/mL streptomycin, and 10% fetal bovine serum (FBS, BI, United States). The medium was replaced every 3 days. The cells were passaged once they reached confluence. These cells progress through three to four growth stages. Fourth-generation ADSCs, which were obtained from different individuals, contained the cells required for exosome extraction.

### 2.2 Adipogenic and osteogenic differentiation of ADSCs

Following prior methods, ADSCs at passage three were divided into osteogenic and adipogenic lineages. Briefly, ADSCs were grown for 2 weeks in a complete osteogenic medium supplemented with 10 mM b-glycerophosphate (Sigma), 0.1 mM dexamethasone (Sigma), and 0.05 mM ascorbic acid (Sigma). Differentiated cells were then stained with alizarin red. ADSCs were grown for 3 weeks and exhibited an adipogenic phenotype when stained with Oil Red O after adipogenic induction.

### 2.3 Characterization of ADSCs

In passage three, ADSCs were identified following earlier protocols. Flow cytometric examination of the cell immunophenotype verified the presence of ADSCs. The ADSC surface markers examined included CD29, CD34, CD44, CD45, CD14, and CD105, which were all PE-labeled.

### 2.4 Concentration and characterization of ADSC-Exos

Exosomes were purified as previously described. Adipose-derived stem cells from the fourth passage were fused, and the cells were then moved to a medium supplemented with serum-free DMEM for 48 h at 37°C in a 5% CO_2_ atmosphere. The media underwent a series of centrifugation procedures after the incubation time. The entire centrifugation process was performed at 4°C, and the initial centrifugation was performed at 300 × g for 10 min. Afterward, the supernatant was centrifuged for 10 min at 1,000 × g and 30 min at 10,000 × g. The supernatant was centrifuged at an ultrahigh pressure for 70 min at 100,000 × g. The precipitate was then placed in PBS to resuspend the pellet made up of ADSC-Exos and stored at −80°C refrigerated for later use after the final ultracentrifugation (100,000 × g for 70 min).

For the identification of ADSC-Exos, 1.0 × 10^9^ vesicles were used. Using a transmission electron microscope, the ultrastructure of ADSC-Exos was examined. The particle dispersion size was analyzed by nanoparticle tracking analysis (NTA) and Nanosight LM10 (Malvern et al., United Kingdom). The expression of the common marker proteins for exosomes CD63 (Abmart, M051014, CHINA), TSG101 (Abmart, T55985, CHINA), and CD81 (Abmart, T557425, CHINA) were examined by Western blot.

### 2.5 Exosome uptake assay

To verify that ADSC-Exos could be internalized by KF, they were tagged with a PKH67 fluorescent cell linker kit (Sigma‒Aldrich, MIDI67-1KT) according to the manufacturer’s instructions. After the nuclei were stained with DAPI (Solarbio, C0065), the labeled ADSC-Exos were cocultured with P3 KF for 24 h, and images were obtained at 0 and 24 h with an Olympus IX81 fluorescence microscope.

### 2.6 Analysis of ferroptosis and fibrosis in keloids

After treatment with Erastin and fer-1, P3 KF were cultured in three groups: the first with a predetermined volume of PBS (control group), the second with Erastin (Erastin group), and the third with fer-1 (fer-1 group). After 24 h, each plate was subjected to Western blot analysis.

### 2.7 Analysis of ferroptosis and fibrosis in keloids cocultured with ADSC-Exos

After coculture with ADSC-Exos, P3 KF were cultured in three groups: the first with a predetermined volume of PBS (control group), the second with ADSC-Exos (ADSC-Exo group), and the third with ADSC-Exos + Erastin (ADSC-Exo + Erastin group). After 24 h, each plate was subjected to Western blot analyses.

### 2.8 Iron metabolism level determination in tissue and cells

Ferroptosis is characterized by free ferrous iron overload and lipid peroxide accumulation. The lipid peroxide (LPO, E-BC-K176-M, Elabscience), reduced glutathione (GSH, A006-1-1, Nanjing, China), and malondialdehyde (MDA E-BC-K027-M, E-BC-K025-M, Elabscience) values of each sample were calculated according to the formula. ROS levels were measured in a medium supplemented with the fluorescent probe DCFH-DA (Solarbio, Shanghai, China) for 20 min at 37°C. A Nikon confocal microscope was used to capture the images.

### 2.9 Western blot analysis

Total protein was extracted in RIPA (Bryotime, P0013B) lysis buffer with a loading buffer (Solarbio, Beijing, China) containing a 1% protease inhibitor cocktail (Solarbio, Beijing, China) and a 1% protein phosphatase inhibitor combination (Solarbio, Beijing, China). The protein concentration was measured with an Instant BCA assay kit (Beyotime, Beijing, China). Twenty micrograms of protein samples were separated by 12.5% and 7.5% sodium dodecyl sulfate-polyacrylamide gel electrophoresis and transferred to polyvinylidene fluoride membranes. At 25°C, the membranes were blocked in 5% nonfat milk in TBST solution for 60–80 min. COLIA1 (A1352, 1:500, ABclonal, United States), COLIIIA1 (ab184993, 1:1,000, Abcam, MA, United States), α-SMA (53-9760-82, 1:1,000, Thermo Fisher, MA, United States), GPX4 (ab125066, 1:1,000, Abcam, MA, United States ), and SLC7A11 (ab175186, 1:1,000, Abcam, MA, United States) were added to the membranes. The protein bands were visualized using a BeyoECL Plus kit (Beyotime, 0018 M, China). Relative gene expression was determined using ImageJ software (http://rsb.info.nih.gov/ij/), with GAPDH (AF7021, 1:3,000, Affinity, United States), β-actin (AF70181:1,000, Affinity, United States), tubulin (M20005, 1:1,000, Abmart, China) or vinculin (T40106, 1:500, Abmart, China) used as the internal loading proteins for normalization.

### 2.10 qRT-PCR assay

TRIzol reagent (TaKaRa) was used to extract total RNA. cDNA was measured using a NanoDrop spectrophotometer (Thermo Fisher, MA, United States). Using a Roche Transcriptor cDNA Synth. Using a kit (Roche, GERMAN), 200 ng of RNA was reverse-transcribed into first-strand cDNA. The FastStart Universal SYBR Green Master Mix (Rox) (Roche GERMAN.) was then used on a Step One Plus Real-time PCR System (Applied Biosystems, Carlsbad, CA, United States). The internal loading of mRNA was performed with β-actin and GAPDH; the fold change in gene expression was computed using the 2^−ΔΔCT^ method. A PCR array (wc-Mrna0271-H) was used to determine which mRNAs related to ferroptosis in KF were affected by ADSC-Exos.

### 2.11 A nude mouse model was established

Twenty-four 4-week-old nude mice (20 ± 5 g) purchased from Harbin Medical University were individually maintained in conventional animal rooms with free access to chow and water. After 3 days of adaptation, sliced fresh keloid tissue (1 cm^3^) was embedded in nude mouse dorsalis, as previously reported. The mice were first injected intraperitoneally (IP) with 50 mg/kg pentobarbital and 10 mg/kg xylazine. The incision was sutured with suture-free glue. In subsequent procedures, the mice were separated into three groups, with 8 mice in each group until 28 d, when the tissue was stable. Then, 200 μg of ADSC-Exos and ADSC-Exos + Erastin (1.25 mg) dissolved in PBS, as well as an equal volume of PBS solution, was injected into the interior of the keloids and injected radially into the backs of the nude mice; this process was repeated every 3 days.

Histological analysis of the mice was terminated 21 days after keloid implantation with an overdose of sodium pentobarbital (150 mg/kg, i.H.). Keloid tissue was fixed in 10% formalin at 4°C, and gradient dehydration was performed using ethanol. The tissue was embedded in paraffin wax and sectioned. HE and Masson’s trichrome staining kits (Solarbio, Beijing, China) were used to assess tissue fibrosis. After staining, the sections were photographed with an attached digital camera and examined microscopically (Olympus, Japan). ImageJ software was used for quantitative analysis. LPO and MDA were used to calculate the levels of lipid peroxides and metabolites in keloid tissues from each group. The GSH content was a significant component for assessing the antioxidant capacity of each group.

After being washed with various ethanol concentrations, Keloid explant specimens have been deparaffinized and rehydrated with xylene. Antigen retrieval was conducted by microwaving the sections in an antigen retrieval solution for 10 min. Sections were cleaned and shaded, incubated in 3% hydrogen peroxide for 10 min at room temperature, followed by 10% goat serum for 10 min. Subsequently, antibodies against collagen I, collagen III, α-SMA, and GPX4 that segment were applied to the slides at a dilution of 1:50 with PBS. The slices were nuclear staining with DAPI (H-1200, Vector Laboratories, Burlingame, CA, United States) after being incubated for 1 h at room temperature with a secondary antibody solution. All slides were counterstained with hematoxylin (Cat. No.H8070, Solarbio, China) and imaged with a microscope.

### 2.12 siRNA transfection

SLC7A11-targeting siRNA and scrambled control siRNA were purchased from Gene Pharma. Lipofectamine 3000 (Invitrogen) was used to transfect the cells with siRNA according to the manufacturer’s instructions. KF were seeded in 6 healthy plates, followed by transfection with 10 nM SLC7A11-targeting siRNA (si-SLC7A11) or nc-RNAi control (si-NC) for 24, 48, or 72 h. The transfection efficiency was determined by observing the position of liposomes under a fluorescence microscope. The transfection efficacy was assessed by examining the position of liposomes under a fluorescence microscope. The results are represented as a percentage of the absorbance ratio between treated and control cells during qRT-PCR verification of gene knockout status.

### 2.13 Statistics

GraphPad Prism 9 (GraphPad Inc., La Jolla, CA, United States) was used for data analysis. Data from three or more experiments were collected and are presented as the mean ± SEM. Student’s *t*-test was used to determine the significance of the differences between the two groups. Three or more independent control and experimental samples were evaluated using one-way ANOVA. A *p*-value < 0.05 indicated statistical significance.

## 3 Results

### 3.1 Keloid fibrosis was induced by ferroptosis

Transmission electron microscopy revealed characteristic changes in ferroptosis in keloid tissues. In this study, we compared the expression of ferroptosis genes in KF and normal fibroblasts. The gene and protein expression levels of GPX4 and GSH in keloids are lower than those in normal skin. Our results showed that lipid peroxide and malondialdehyde levels in tissues or cells from keloids were greater than those in tissues or cells from normal skin. The expression of oxidative stress-related target genes related to ferroptosis, such as PTGS2 (COX2), NRF2, and nuclear factor E2-related factor 2 (Nrf2), was detected (Supplementary Material S1).

**FIGURE 1:**
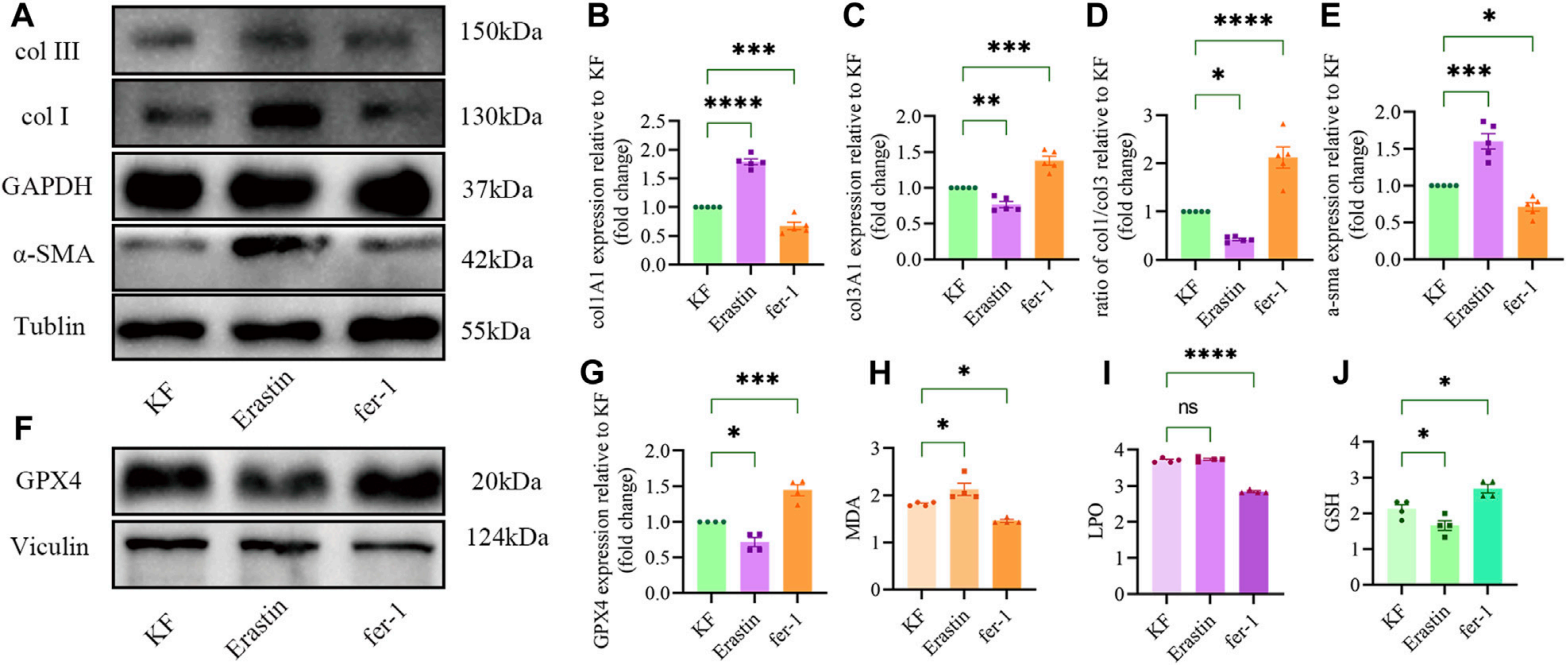
Effects of Erastin and fer-1 on fibrosis and ferroptosis in KF. **(A)** Western blot images of keloid fibrosis affected by Erastin (2.5 μm/mL) and fer-1 (1.7 μm/mL). **(B)** Quantification of the expression of col1A1, **(C)** col3A1, and **(D)** the ratio of col1A1 to col3A1, **(E)** α-SMA relative to keloid fibroblast (KF) (n = 5). **(F)** Western blot images of GPX4 in KF treated with Erastin and fer-1. **(G)** Quantification of GPX4 and **(H)** MDA levels. **(I)** LPO and **(J)** GSH relative to KF. GPX4, glutathione peroxidase 4; MDA, malondialdehyde; LPO, liposomal peroxidation; GSH, glutathione. Four repetitions were carried out for each experiment. The error bars represent the standard deviation. One-way ANOVA followed by Tukey’s multiple comparison test was carried out for comparisons. * represents *p* < 0.05, ** represents *p* < 0.01, *** represents *p* < 0.001, **** represents *p* < 0.0001, ns represents no significance.

Erastin can activate the fibrogenic ability of KF. Increased the expression of type I collagen and α-SMA but decreased that of type III collagen, promoting the ratio of type I collagen to type III collagen in keloid centers, facilitating the formation of fibrosis in keloids (Figures 1A–E).

By activating ferroptosis, Erastin can increase the levels of liposomal peroxidation (LPO) metabolites, increase malondialdehyde (MDA) levels, decrease GSH levels, and decrease the expression of the GPX4 protein. However, fer-1 had the opposite effect on Erastin (Figures 1F–J).

### 3.2 Characterization of ADSCs and ADSC-Exos

**FIGURE 2:**
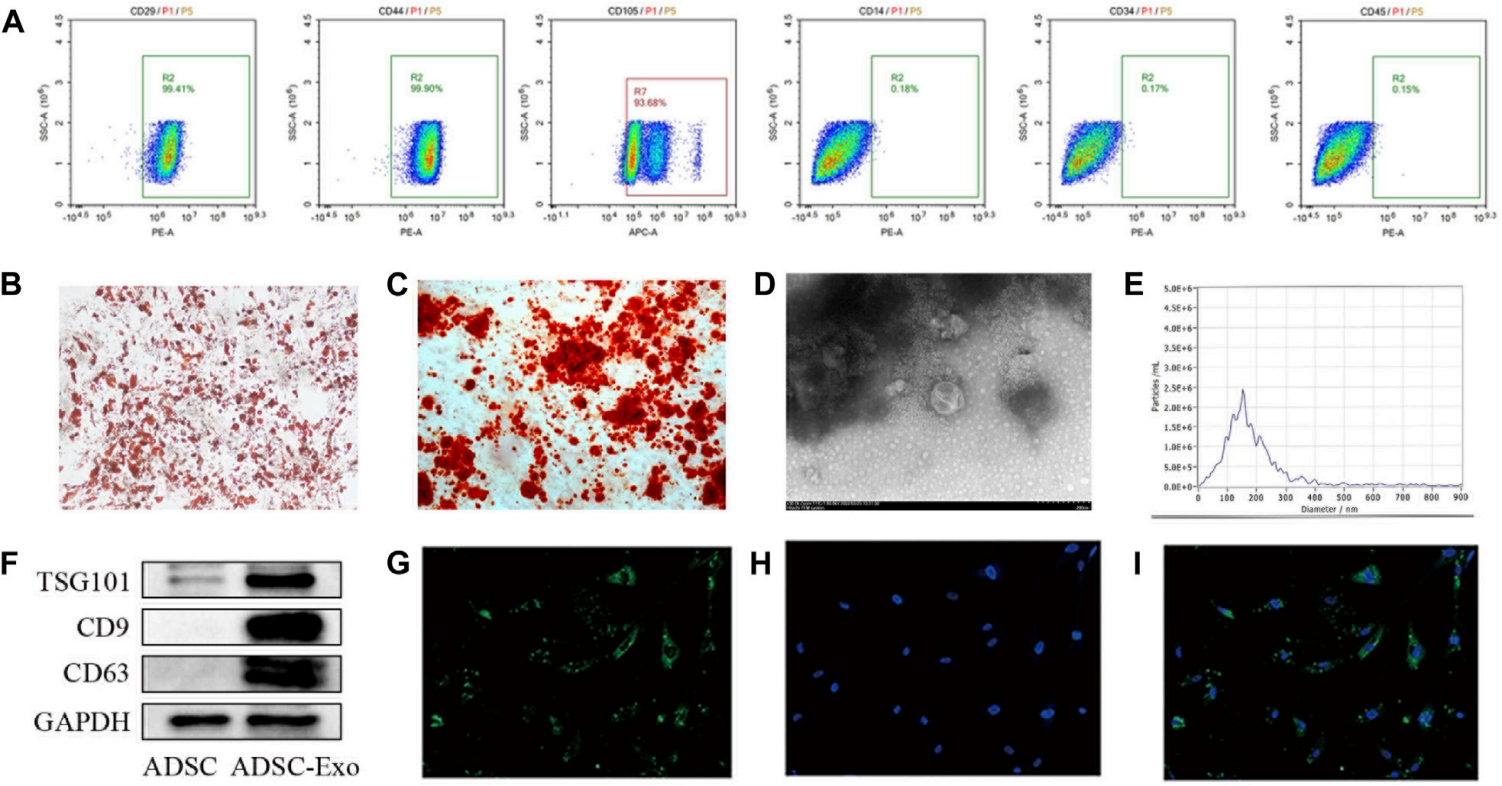
Characteristics of adipose-derived stem cells (ADSCs). **(A)** Results of flow cytometry analysis of ADSCs showing the expression of mesenchymal stem cell surface markers. CD29, CD44, and CD105 were positive; CD14, CD34 and CD45 were negative. **(B)** Adipogenic and **(C)** osteogenic differentiation measured by Oil Red O staining and Alizarin Red S staining; scale bars = 100 μm. **(D)** The morphology of ADSC-Exos was analyzed by TEM; scale bar = 200 nm. **(E)** The particle size of ADSC-Exos was measured by NTA. **(F)** Western blot images of the exosome markers CD9, CD81, and TSC101. **(G–I)** Confocal microscopy was used to observe the internalization of ADSC-Exos labeled with PKH-67 into **(G)** KF, **(H)** DAPI, and **(I)** merged images.

First, the acquired ADSCs were negative for CD45, CD14, and CD45 but positive for the MSC surface indicators CD29, CD44, and CD105. These findings suggested that the ADSCs were suitable for further application (Figure 2A). The capacity of the ADSCs to differentiate into osteoblasts and idioblasts was further validated by alizarin red staining and Oil red O staining, respectively (Figures 2B, C). The particles that were removed from the ADSCs were also identified. The particles were observed to have a characteristic oval shape via TEM (Figure 2D). The NTA results revealed that the sizes of the isolated particles were primarily in the 100–170 nm range (Figure 2E). TSG101, CD9, and CD63 are recognized exosome markers that were further analyzed by Western blot analysis (Figure 2F). ADSC-Exos were tagged with PKH67 and cocultured with KF. After 24 h, ADSC-Exos were removed and delivered to the cytoplasm of KF (Figures 2G–I). These results indicate that ADSC-Exos were effectively separated and transferred to KF.

### 3.3 ADSC-Exos alleviated KF fibrosis by inhibiting ferroptosis

**FIGURE 3:**
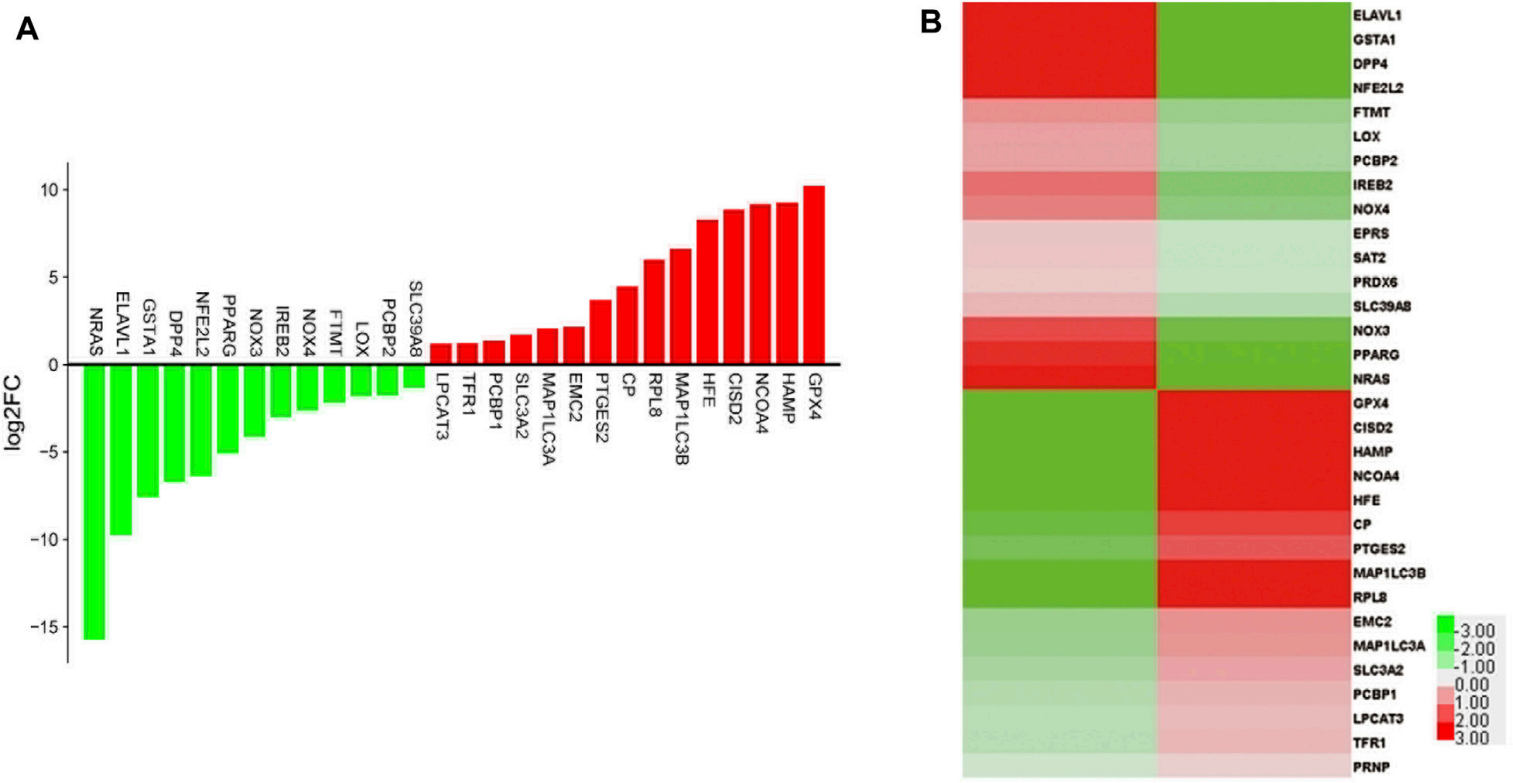
Twenty-eight DEGs were identified in KF cocultured with ADSC-Exos and in the control group by ferroptosis-related qPCR array. **(A)** Histogram and **(B)** heatmap of gene expression in KF cocultured with ADSC-Exos and control cells.

The differential gene expression between the ADSC-Exos and control groups was compared by ferroptosis PCR array. Compared with that in the untreated group, the expression of GPX4, the core gene involved in ferroptosis, was greater (Figure 3). Surprisingly, we discovered that GPX4 was significantly differentially expressed between the keloid and normal skin groups, indicating that ADSC-Exos could be a novel but crucial target for preventing the occurrence and development of KF fibrosis.

**FIGURE 4:**
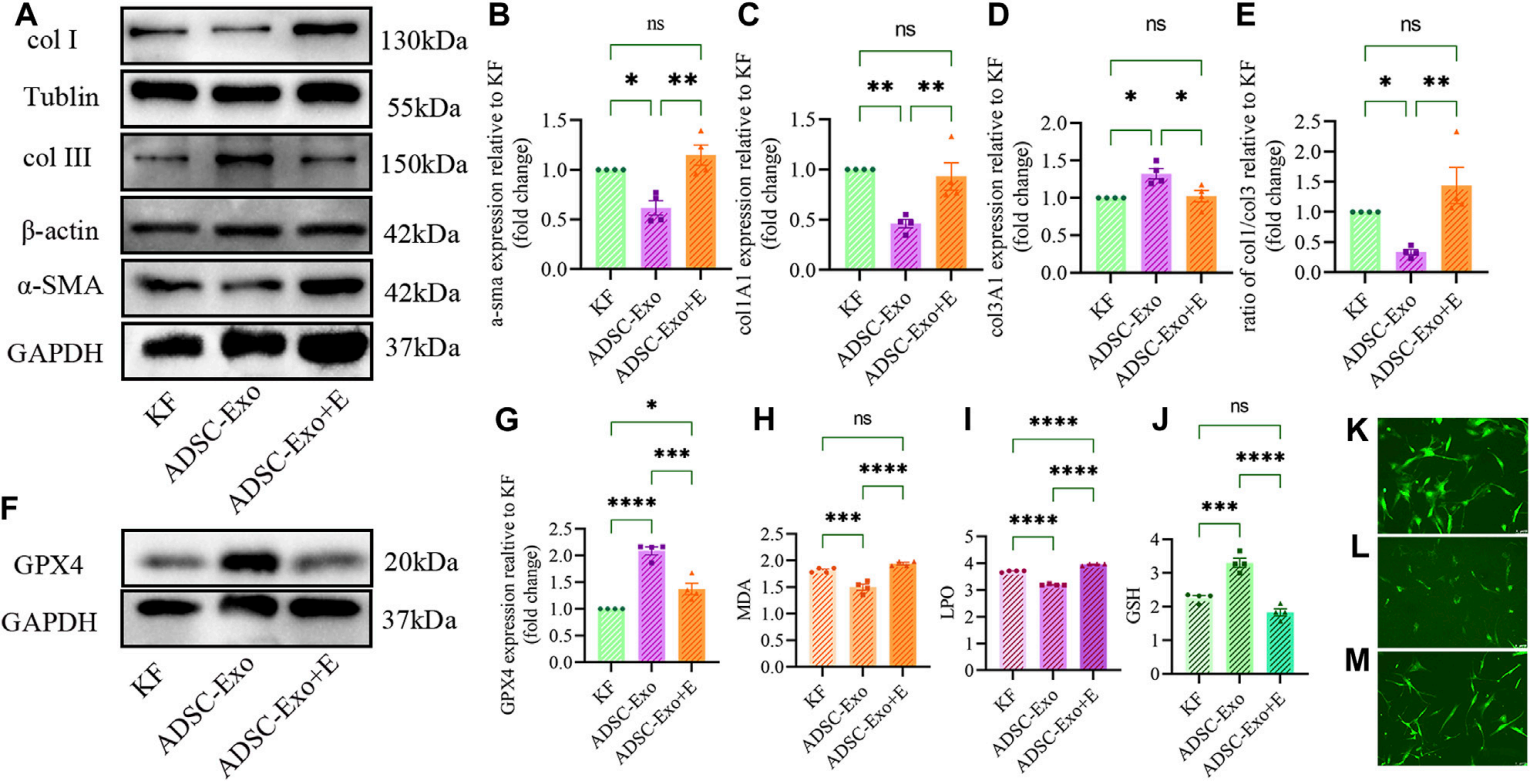
ADSC-Exos upregulated the GPX4-GSH axis and inhibited collagen synthesis and fibronectin *in vitro*. **(A)** Western blot images of fibrosis in KF cocultured with ADSC-Exos and ADSC-Exos + Erastin. **(B)** Quantified expression of α-SMA, **(C)** col1A1, **(D)** col3A1, **(E)** the ratio of col1A1 to col3A1; **(F)** Western blot images of GPX4 in KF cocultured with ADSC-Exos and ADSC-Exos + Erastin. **(G)** Quantification of GPX4 and **(H)** MDA levels. **(I)** LPO and **(J)** GSH relative to KF (n = 4). **(K–M)** Representative images of the detection of ROS. **(K)** KF, **(J)** KF cocultured with ADSC-Exos. **(M)** KF cocultured with ADSC-Exos + Erastin.The data are shown as the mean ± SEM. * represents *p* < 0.05, ** represents *p* < 0.01, *** represents *p* < 0.001, **** represents *p* < 0.0001, ns represents no significance.

ADSC-Exos, which resembles fer-1, can restrain the fibrogenic process of KF, decreasing the expression of type I collagen and α-SMA while decreasing the expression of type III collagen, reducing the ratio of type I collagen to type III collagen in keloids (Figures 4A–E). In contrast, GPX4 protein expression was increased, and KF fibrosis was alleviated by ADSC-Exos. Erastin prevents this therapeutic effect. The accumulation of MDA and LPO, on the other hand, was reduced by ADSC-Exos, increasing the expression of GSH in the KF (Figures 4F–J). The intracellular ROS level was decreased by ADSC-Exos. Erastin prevents all the therapeutic effects of ADSC-Exos by activating ferroptosis (Figures 4K–M).

### 3.4 ADSC-Exos alleviated pathological keloid injury *in vivo*

**FIGURE 5:**
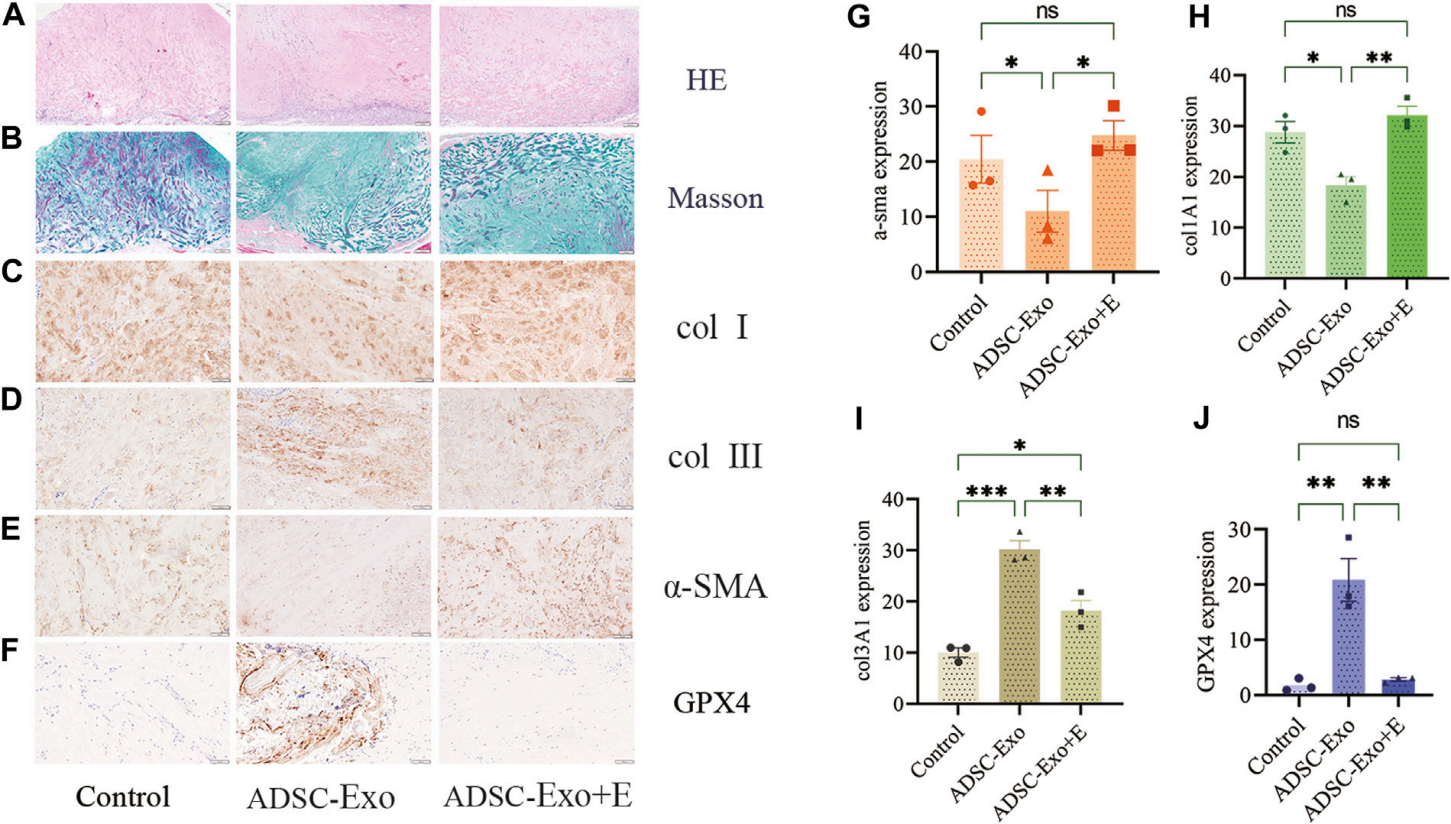
ADSC-Exos upregulated the GPX4 axis and inhibited collagen synthesis and fibronectin *in vivo*. The tissues on the backs of the mice treated with PBS, ADSC-Exos, or ADSC-Exos + Erastin were collected on day 21 postintervention. Typical histological images of keloid tissues stained with **(A)** H&E and **(B)** Masson’s trichrome. Immunohistochemical images of **(C)** col1A1, **(D)** col3A1, **(E)** α-SMA, and **(F)** GPX4. Quantification of the expression of **(G)** col1A1, **(H)** col3A1, **(I)** α-SMA, and **(J)** GPX4 (n = 3). The data are shown as the mean ± SEM. * represents *p* < 0.05, ** represents *p* < 0.01, *** represents *p* < 0.001.

**FIGURE 6:**
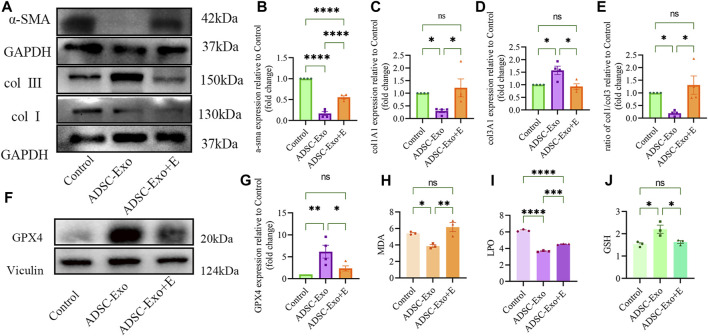
ADSC-Exos upregulated the GPX4 axis and inhibited collagen synthesis and fibronectin *in vivo*. **(A)** Western blot images of the control group, ADSC-Exo group and ADSC-Exos + Erastin group. **(B)** Quantified expression of α-SMA, **(C)** col1A1, **(D)** col3A1, **(E)** the ratio of col1A1 to col3A1 (n = 4); **(F)** Western blot images of GPX4 of the control group, ADSC-Exo group and ADSC-Exos + Erastin group. **(G)** Quantification of GPX4 (n = 4) and **(H)** MDA levels. **(I)** LPO and **(J)** GSH relative to KF (n = 3).

Using a nude mouse keloid model, we investigated the therapeutic efficacy of ADSC-Exos. The therapeutic impact of ADSC-Exos on keloid pathology was observed using H&E and Masson staining. Figures 5A, B depicts the usual histological alterations of keloids in each model category. After constructing the keloid model, we examined the expression of collagen I, collagen III, α-SMA, and GPX4 after 21 days. Immunohistochemical labeling (Figures 5C–J) confirmed the findings of Western blot analysis (Figure 6): the expression of collagen I and α-SMA and the ratio of collagen I to collagen III were significantly lower in the keloid region treated with ADSC-Exos than in the control group or the ADSC-Exo + Erastin group. Figures 6F–J shows the increase in the expression of GPX4 and GSH and the decrease in the accumulation of LPO and MDA induced by ADSC-Exos *in vivo*. However, Erastin impeded the effect of ADSC-Exos.

### 3.5 ADSC-Exos inhibits fibrosis in keloids by promoting SLC7A11-GPX4 *in vitro*

**FIGURE 7:**
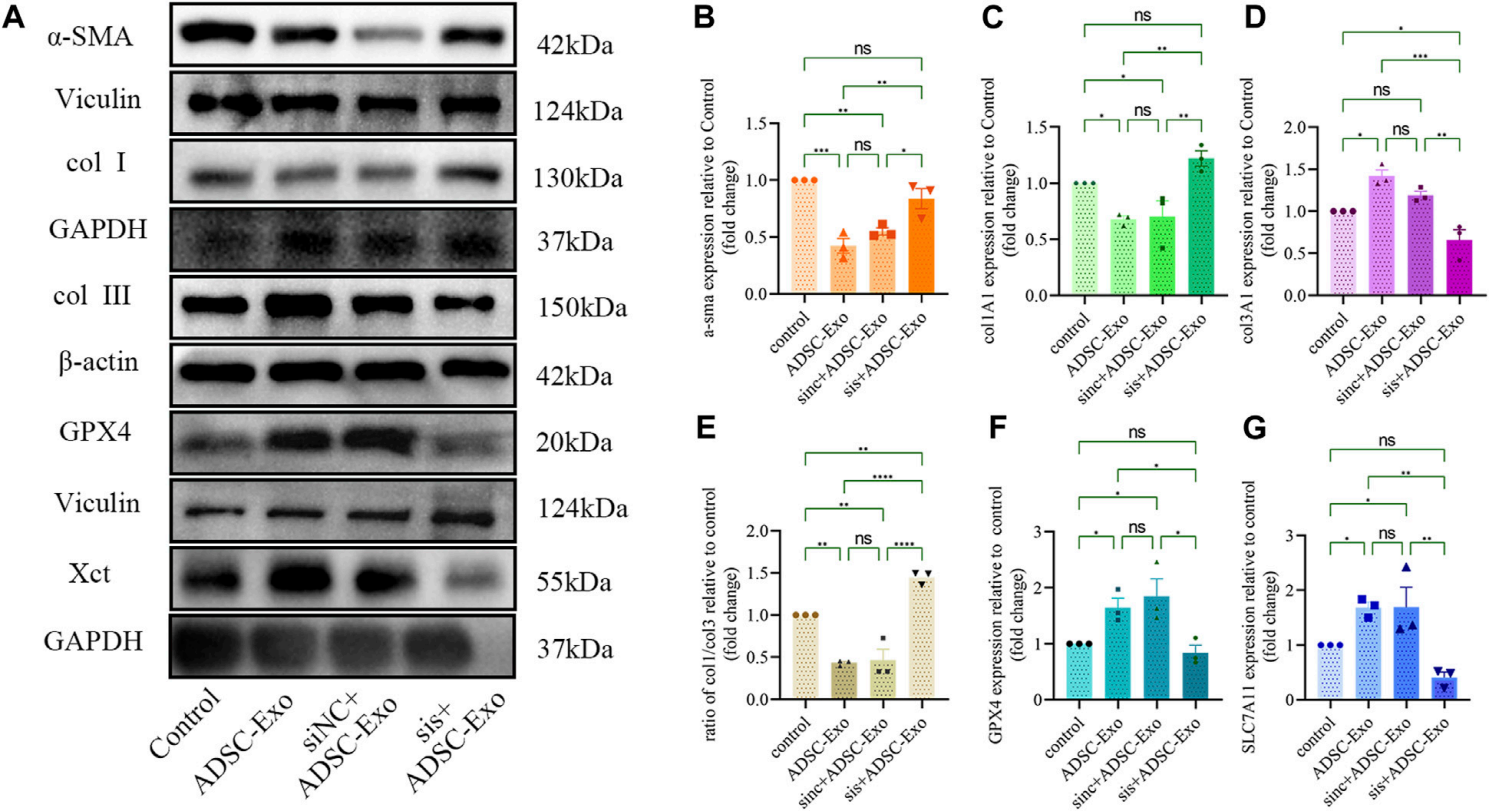
ADSC-Exos upregulated the SLC7A11-GPX4-GSH axis and inhibited collagen synthesis, fibronectin, and ferroptosis-related genes *in vitro*. **(A)** Western blot images of the control group, ADSC-Exo group, sinc + ADSC-Exo group, and sis + ADSC-Exo group. **(B)** Quantification of the expression of **(B)** α-SMA, **(C)** col1A1, **(D)** col3A1, **(E)** the ratio of col1A1 to col3A1, **(F)** GPX4, and **(G)** SLC7A11 relative to the control (n = 3). The data are shown as the mean ± SEM. * represents *p* < 0.05, ** represents *p* < 0.01, *** represents *p* < 0.001, **** represents *p* < 0.0001, ns represents no significance.

To further explore the role of ADSC-Exos in keloid fibrosis, we investigated how ADSC-Exos protects against keloid iron sagging. Here, more attention has been given to the regulation of the SLC7A11-GPX4 pathway. ADSC-Exos were cocultured with SLC7A11-silenced KF. Compared with those of the controls, ADSC-Exos and ADSC-Exos cocultured with si-NC KF inhibited the expression of fibrosis-related genes in keloids and increased the protein expression of SLC7A11 and GPX4. At the same time, SLC7A11 silenced ADSC-Exos and antagonized this effect (Figure 7).

## 4 Discussion

Keloids are pathological scars with a high incidence rate. It can not only cause pain and itching but also affect the patient’s mental state and quality of life. In severe cases, it can even affect affected limb function (Fu et al., 2024). Therefore, studying the mechanism underlying the formation and prevention of pathological scars is a hot topic in the medical field (Xu et al., 2022). Additionally, exploring a treatment method that can target the pathogenesis of keloids is the key to solving this problem.

Emerging research suggests that ferroptosis can modulate fibrosis (Qiu et al., 2024). Several genes influence keloid development. Although there are differences in gene expression between keloid and normal skin fibroblasts, the exact etiology of iron deficiency remains unexplained. In this study, we compared the expression of ferroptosis genes and metabolic products of iron in keloid and normal skin. The results showed that keloid fibrosis was associated with a reduction in GPX4 and GSH, which could not prevent the accumulation of lipid metabolite products during ferroptosis progression in keloids. Our research provides some evidence confirming the relationship between ferroptosis and the potential mechanism of keloid formation.

Exosomes from human adipose-derived mesenchymal stem cells can lower the activation of the fibrosis signaling system by preventing myofibroblast formation and increasing the level of transforming growth factor. Furthermore, by activating the ERK/MAPK pathway, ADSC-Exos increases the expression of matrix metalloproteinase-3 (MMP3) in dermal fibroblasts, resulting in a high ratio of MMP3 to tissue inhibitor of matrix metalloproteinase-1 (TIMP1), which is conducive to extracellular matrix (ECM) remodeling (Wang et al., 2017). The number of myofibroblasts increases during keloid repair. α-SMA is a myofibroblast differentiation marker that promotes myofibroblast release and wound healing. In our study, the exosomes of adipose-derived stem cells decreased the levels of α-SMA and collagen I and the ratio of type I to III collagen. Keloid is a dermal fibroproliferative tumor that can be recognized by excessive ECM accumulation. The ratio of Col1/Col3 is believed to improve in the later stage of ECM reshaping (Peeters et al., 2014). The ratio of type I to III collagen in fibroblasts in keloid tissue was greater than that in normal skin (*p* < 0.05) (Zhang et al., 2009). Collagen I is a stiff fibrillar protein that provides tensile strength (You et al., 2023), whereas collagen III forms an elastic network and stores elastic rebound kinetic energy (Wang et al., 2022). Our findings suggest that ADSC-Exos can transform thick and stiff collagen fibers into slender and elastic fibers in the dermis, which is likely to promote the development of keloids into normal skin. However, more research needs to be conducted on this topic.

Like fer-1, ADSC-Exos decreased fibrosis via ferroptosis in KF, decreased lipid peroxidation, and increased GPX4 and GSH expression. Erastin can promote ferroptosis in keloids and decrease the functionality of ADSC-Exos, accompanied by excessive fibrosis. These results suggest a new possible mechanism by which ADSC-Exos inhibits the myofibroblast differentiation of KF by decreasing ferroptosis in keloids.

The activity of GPX4 is dependent on the activation of the cystine transporter SLC7A11 (Bayir et al., 2023; Wang H. et al., 2023). We hypothesized that the increase in GPX4 signaling in keloids may be mediated by exosomes that increase the expression of SLC7A11 in keloids. By knocking out SLC7A11 in KF, the anti-ferroptosis or anti-fibrosis effects of ADSC-Exos were antagonized. Our results indicate that ADSC-Exos are involved in inhibiting myofibroblast differentiation and collagen production in KF by activating the SLC7A11-GPX4 signaling pathway to reduce ferroptosis.

The limitation of our study is that although an increase in SLC7A11-GSH-GPX4 was observed in KF treated with ADSC-Exos, it was not easy to detect the exact type of lipid. In subsequent studies, we combined ferroptosis-related oxidative lipidomics and keloids and further explored lipid metabolism after treatment with ADSC-Exos, accelerating the clinical transformation of ADSC-Exos.

In conclusion, iron metabolism disorder-induced ferroptosis is involved in the pathogenesis and persistent activation of myofibroblasts in KF. ADSC-Exos regulates GPX4 in KF and suppresses keloid fibrosis *in vitro* via SLC7A11-GPX4. By suppressing keloids, ADSC-Exos, and ferroptosis can become viable therapeutic targets.

## 5 Conclusion

Restraining ferroptosis can enhance the anti-fibrotic effect of keloid cells. ADSC-Exos can significantly reduce the degree of fibrosis in keloids by inhibiting ferroptosis. By regulating the occurrence of the SLC7A11-GPX4 signaling pathway, it can inhibit ferroptosis in keloid cells, thereby reducing fibrosis.

## Data Availability

The raw data supporting the conclusions of this article will be made available by the authors, without undue reservation.
