# Correction: Human adipose mesenchymal stem cell-derived exosomes alleviate fibrosis by restraining ferroptosis in keloids

**DOI:** 10.3389/fphar.2025.1705356

**Published:** 2025-10-01

**Authors:** Yuan Tian, Meijia Li, Rong Cheng, Xinyue Chen, Zhishan Xu, Jian Yuan, Zhiyong Diao, Lijun Hao

**Affiliations:** Plastic Surgery, Harbin Medical University, Harbin, China

**Keywords:** adipose-derived mesenchymal stem cells, fibrosis, ferritic, extracellular vesicles, GPx4

Author **Lijun Hao** was erroneously assigned as the sole corresponding author. Author **Zhiyong Diao** is also a corresponding author.

Authors **Lijun Hao** and **Zhiyong Diao** were erroneously omitted as equal contributing authors.

The original article has been updated.

