# Supplementary figures and images for: Human adipose mesenchymal stem cell-derived exosomes alleviate fibrosis by restraining ferroptosis in keloids
Source: Front Pharmacol. 2024 Aug 16;15:1431846. doi: 10.3389/fphar.2024.1431846 (PMC11361945; doi:10.3389/fphar.2024.1431846)

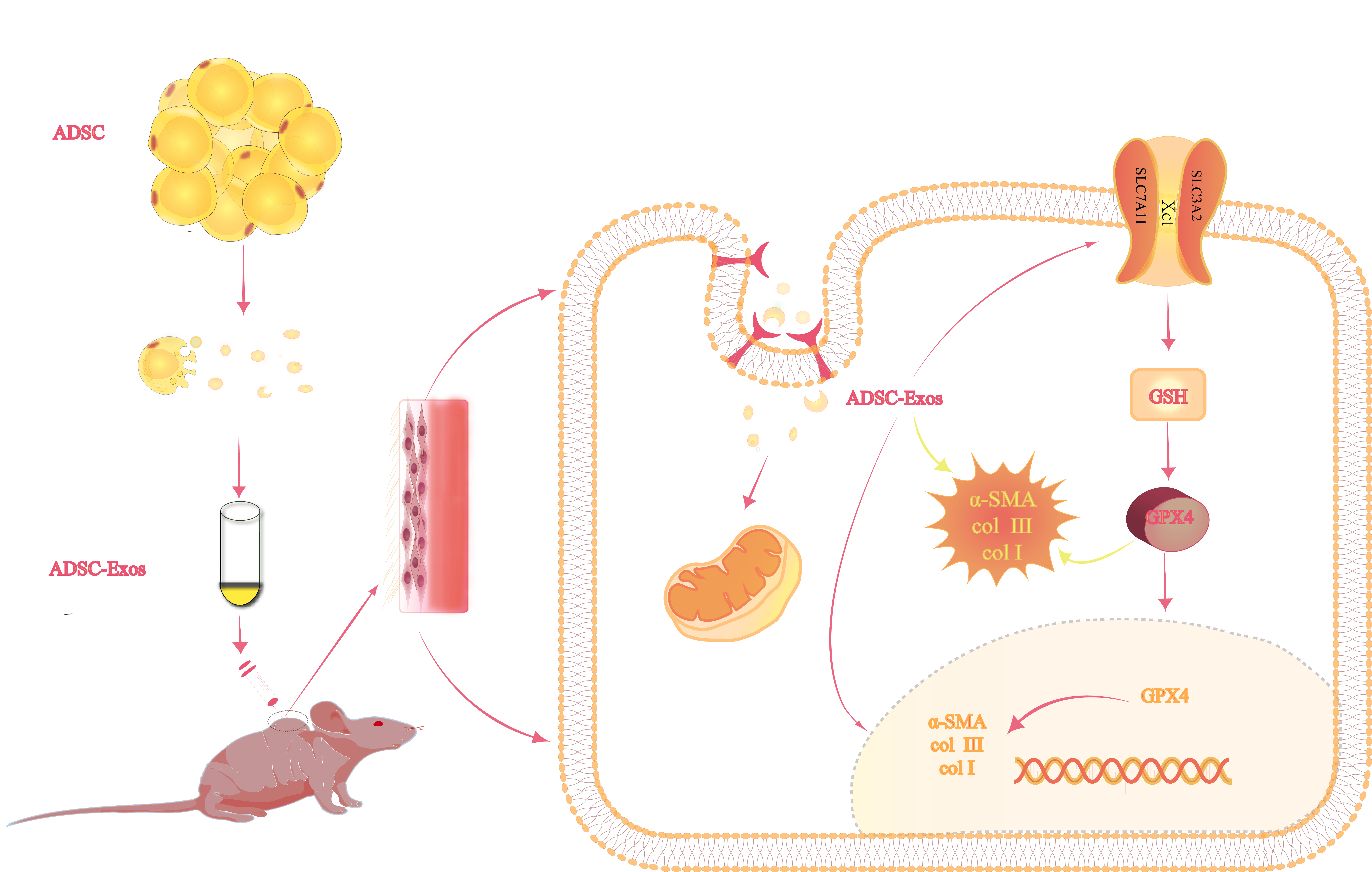

Supplement: Supplementary file 1 [file Image2.JPEG]

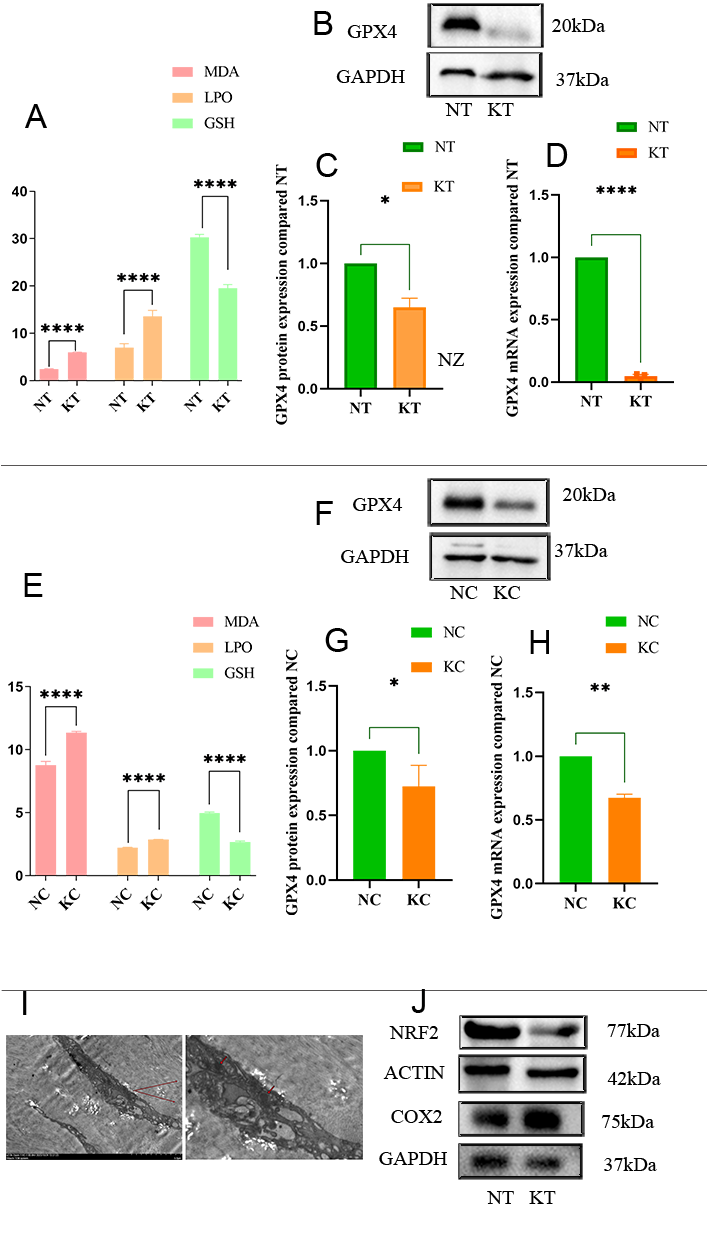

Supplement: Supplementary file 2 [file Image1.TIF]

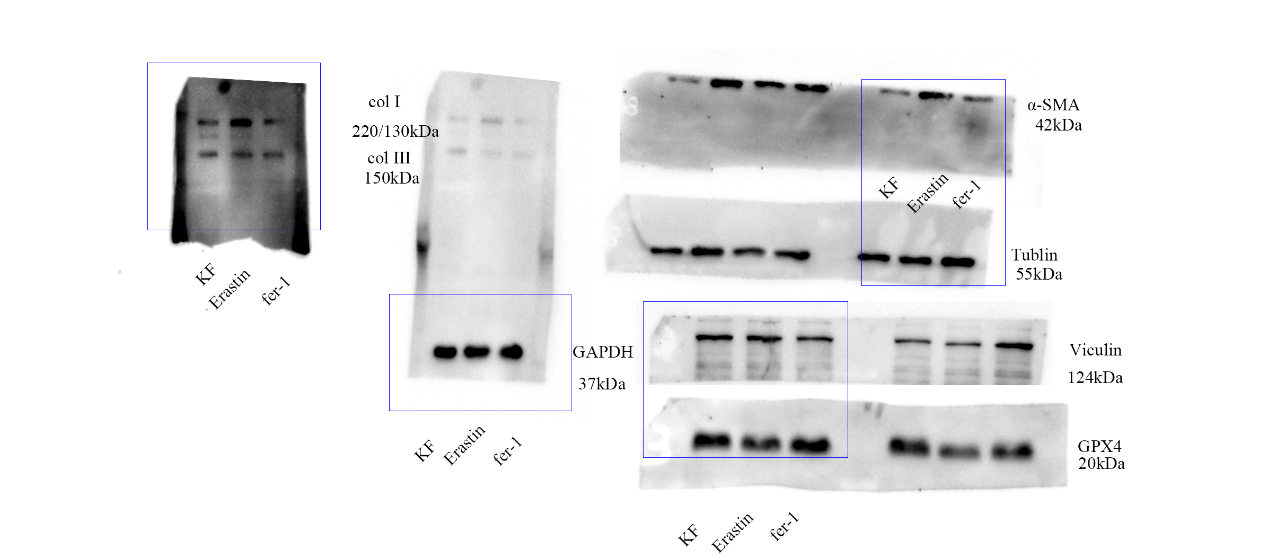


FIG.1


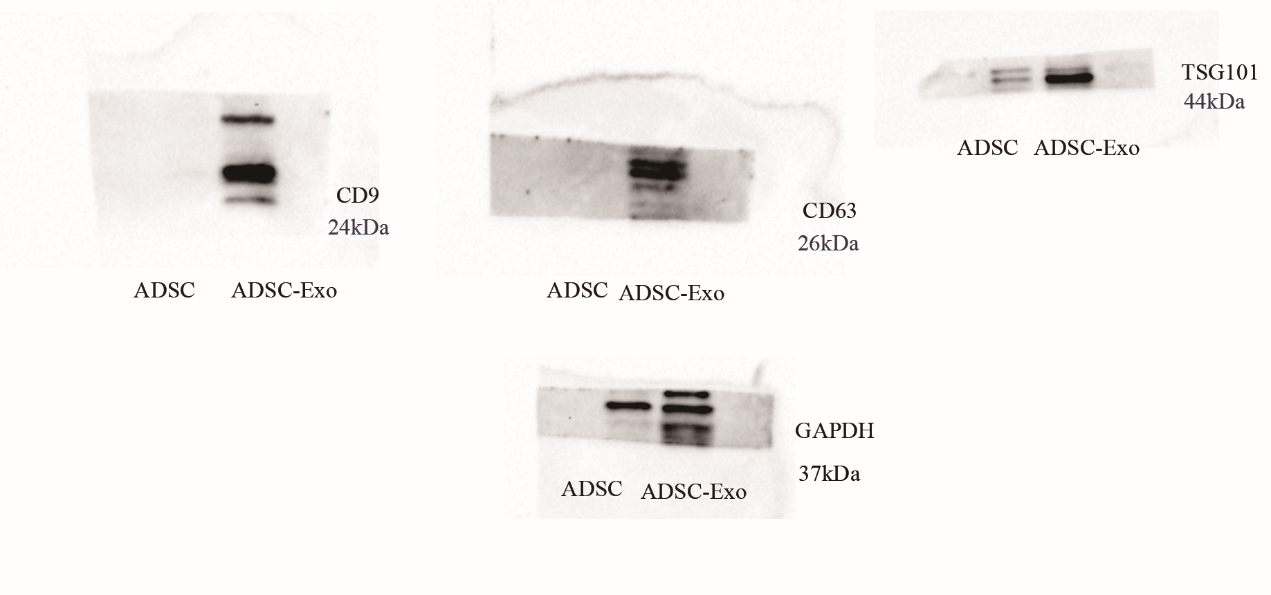


FIG.2


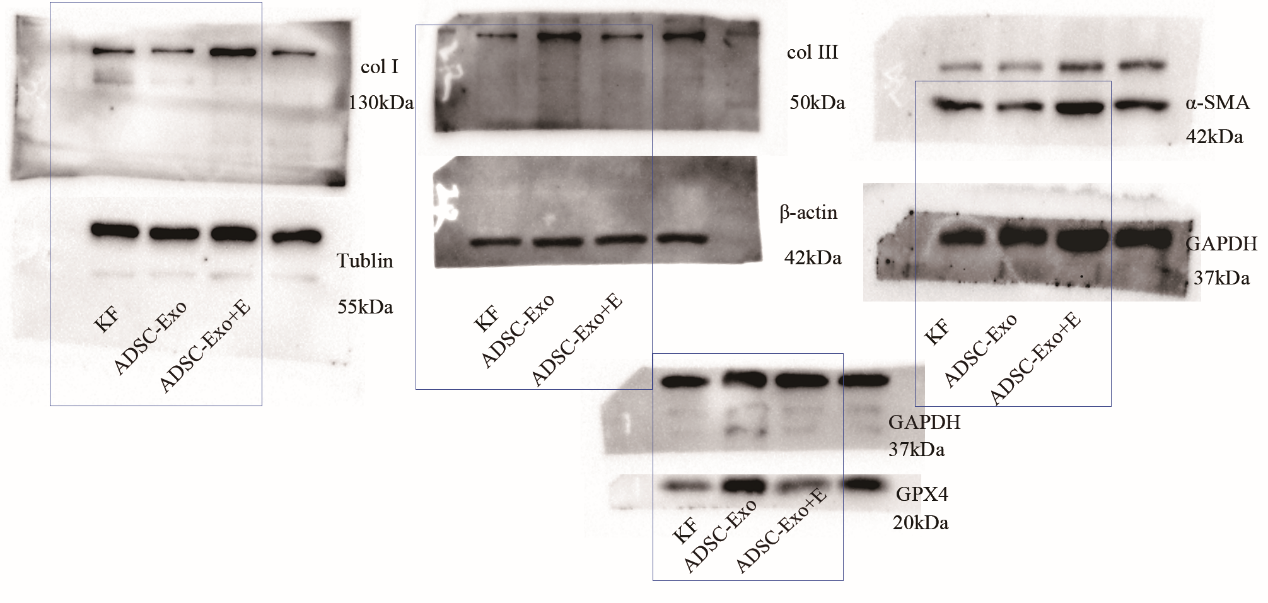


FIG.4


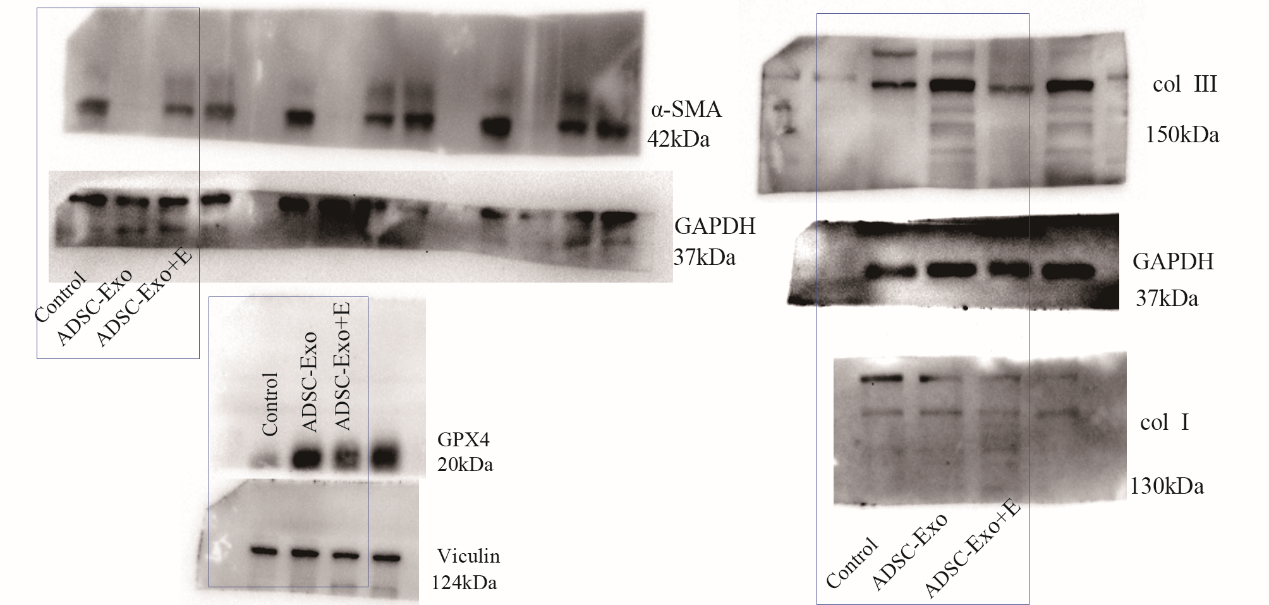


FIG.6


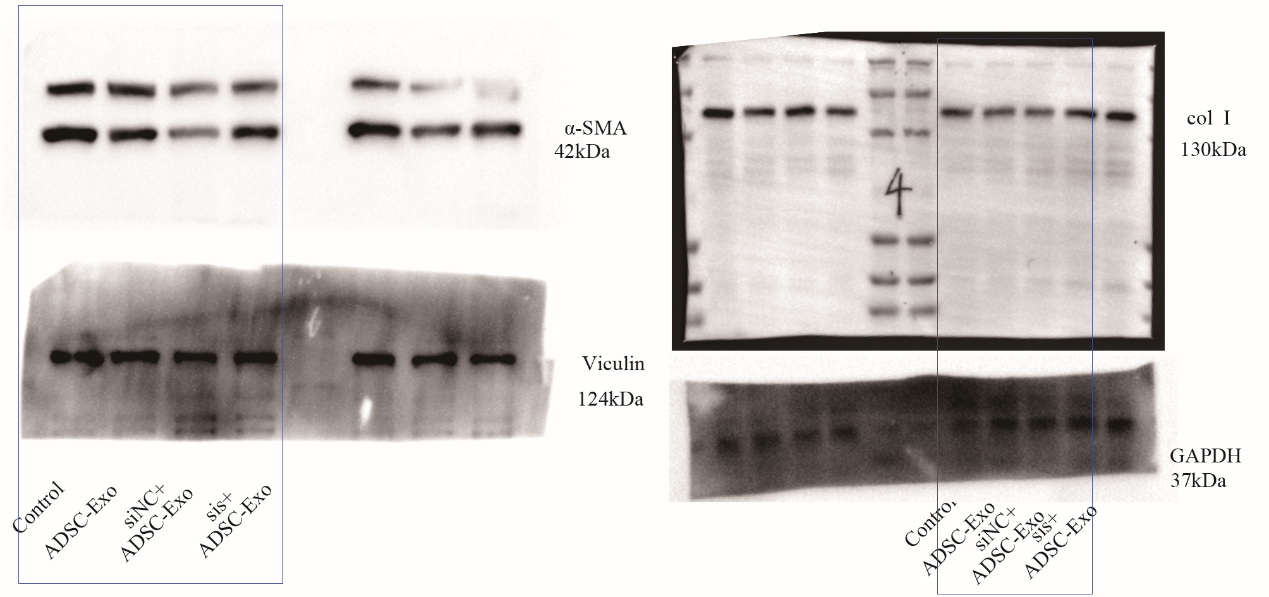


FIG.7-1


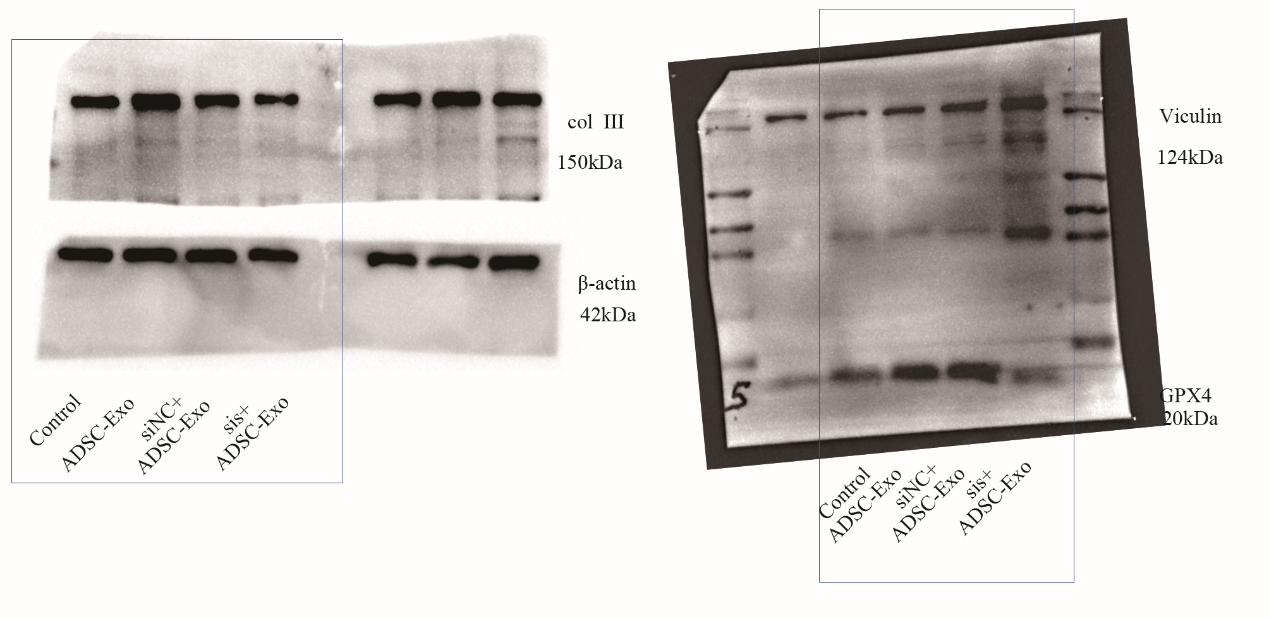


FIG.7-2


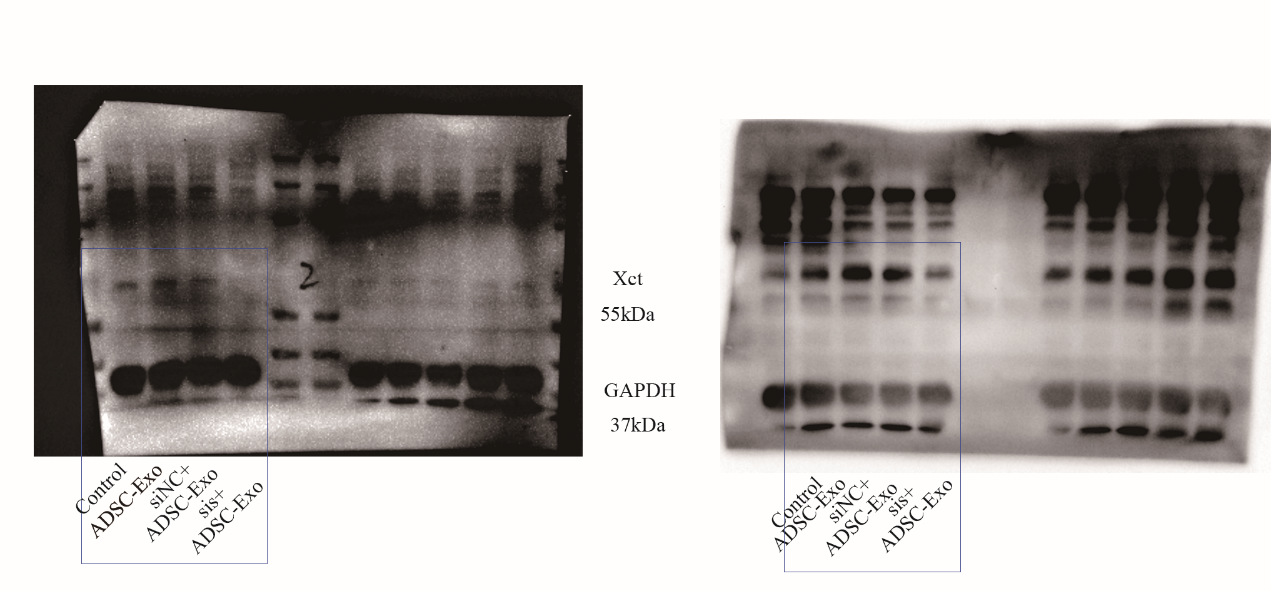


FIG.7-3

Supplement: Supplementary file 3 [file DataSheet2.docx]
