# Supplementary material for: Human adipose mesenchymal stem cell-derived exosomes alleviate fibrosis by restraining ferroptosis in keloids
Source: Front Pharmacol. 2024 Aug 16;15:1431846. doi: 10.3389/fphar.2024.1431846 (PMC11361945; doi:10.3389/fphar.2024.1431846)
Supplement: Supplementary file 4 [file DataSheet1.docx]

| Fig1-B-α-SMA | | | | | | |  | |  |  |  |
| --- | --- | --- | --- | --- | --- | --- | --- | --- | --- | --- | --- |
| Bonferroni's multiple comparisons test | Mean Diff. | 95.00% CI of diff. | Below threshold? | Summary | Adjusted P Value |  | |  |  |  |  |
| KF vs. Erastin | -0.6018 | -0.8768 to -0.3269 | Yes | *** | 0.0002 | A-B | |  |  |  |  |
| KF vs. fer-1 | 0.2908 | 0.01583 to 0.5657 | Yes | * | 0.0372 | A-C | |  |  |  |  |
| Erastin vs. fer-1 | 0.8926 | 0.6177 to 1.168 | Yes | **** | <0.0001 | B-C | |  |  |  |  |
| Fig1-C-COL1A1 | | | | | | | |  |  |  |  |
| KF vs. Erastin | -0.7913 | -0.9625 to -0.6200 | Yes | **** | <0.0001 | B | |  |  |  |  |
| KF vs. fer-1 | 0.3266 | 0.1553 to 0.4978 | Yes | *** | 0.0008 | C | |  |  |  |  |
| Fig1-D-COL3A1 | | | | | | | |  |  |  |  |
| KF vs. Erastin | 0.2352 | 0.06240 to 0.4080 | Yes | ** | 0.0078 | A-B | |  |  |  |  |
| KF vs. fer-1 | -0.3782 | -0.5510 to -0.2054 | Yes | *** | 0.0002 | A-C | |  |  |  |  |
| Erastin vs. fer-1 | -0.6134 | -0.7862 to -0.4406 | Yes | **** | <0.0001 | B-C | |  |  |  |  |
| Fig1-E-COL1A1/ COL3A1 | | | | | | | |  |  |  |  |
| KF vs. Erastin | 0.5720 | 0.1057 to 1.038 | Yes | * | 0.0171 | B | |  |  |  |  |
| KF vs. fer-1 | -1.121 | -1.587 to -0.6547 | Yes | **** | <0.0001 | C | |  |  |  |  |
| Fig1-G-GPX4 | | | | | | | |  |  |  |  |
| KF vs. Erastin | 0.2859 | 0.06706 to 0.5048 | Yes | * | 0.0133 | B | |  |  |  |  |
| KF vs. fer-1 | -0.4445 | -0.6633 to -0.2257 | Yes | *** | 0.0008 | C | |  |  |  |  |
| Fig1-H-MDA | | | | | | | |  |  |  |  |
| KF vs. Erastin | -0.3093 | -0.6046 to -0.01405 | Yes | * | 0.0406 | B | |  |  |  |  |
| KF vs. fer-1 | 0.3600 | 0.06473 to 0.6552 | Yes | * | 0.0193 | C | |  |  |  |  |
| Fig1-I-LPO | | | | | | | |  |  |  |  |
| KF vs. Erastin | -0.03031 | KF vs. Erastin |  | ns | 0.4673 | B | |  |  |  |  |
| KF vs. fer-1 | 0.8649 | KF vs. fer-1 | Yes | **** | <0.0001 | C | |  |  |  |  |
| Fig1-J-GSH | | | | | | | |  |  |  |  |
| KF vs. Erastin | 0.4634 | KF vs. Erastin | Yes | * | 0.0147 | B | |  |  |  |  |
| KF vs. fer-1 | -0.5714 | KF vs. fer-1 | Yes | * | 0.0284 | C | |  |  |  |  |
|  | Mean Diff. | Below threshold? | Summary | Adjusted P Value |  |  | |  |  |  |  |
| Fig4-B-α-SMA | | | | | | | |  |  |  |  |
| KF vs. ADSC-Exo | 0.3834 | Yes | * | 0.0393 | A-B |  | |  |  |  |  |
| KF vs. ADSC-Exo+E | -0.1473 | No | ns | 0.7368 | A-C |  | |  |  |  |  |
| ADSC-Exo vs. ADSC-Exo+E | -0.5307 | Yes | ** | 0.0017 | B-C |  | |  |  |  |  |
| Fig4-C-COL1A1 | | | | | | | |  |  |  |  |
| KF vs. ADSC-Exo | 0.5376 | Yes | ** | 0.0037 | A-B |  | |  |  |  |  |
| KF vs. ADSC-Exo+E | 0.06948 | No | ns | 0.5639 | A-C |  | |  |  |  |  |
| ADSC-Exo vs. ADSC-Exo+E | -0.4681 | Yes | ** | 0.0059 | B-C |  | |  |  |  |  |
| Fig4-D-COL3A1 | | | | | | | |  |  |  |  |
| KF vs. ADSC-Exo | -0.3212 | Yes | * | 0.0130 | A-B |  | |  |  |  |  |
| KF vs. ADSC-Exo+E | -0.02320 | No | ns | 0.7909 | A-C |  | |  |  |  |  |
| ADSC-Exo vs. ADSC-Exo+E | 0.2980 | Yes | 0.0132 | B-C |  |  | |  |  |  |  |
| Fig4-E-COL1A1/ COL3A1 | | | | | | | |  |  |  |  |
| KF vs. ADSC-Exo | 0.6633 | Yes | * | 0.0122 | A-B |  | |  |  |  |  |
| KF vs. ADSC-Exo+E | -0.4395 | No | ns | 0.1226 | A-C |  | |  |  |  |  |
| ADSC-Exo vs. ADSC-Exo+E | -1.103 | Yes | ** | 0.00689 | B-C |  | |  |  |  |  |
| Fig4-G-GPX4 | | | | | | | |  |  |  |  |
| KF vs. ADSC-Exo | -1.087 | Yes | **** | <0.0001 | A-B |  | |  |  |  |  |
| KF vs. ADSC-Exo+E | -0.3744 | Yes | * | 0.0161 | A-C |  | |  |  |  |  |
| ADSC-Exo vs. ADSC-Exo+E | 0.7129 | Yes | *** | 0.0002 | B-C |  | |  |  |  |  |
| Fig4-H-MDA | | | | | | | |  |  |  |  |
| KF vs. ADSC-Exo | 0.3182 | Yes | *** | 0.0006 | A-B |  | |  |  |  |  |
| KF vs. ADSC-Exo+E | -0.1243 | No | ns | 0.1273 | A-C |  | |  |  |  |  |
| ADSC-Exo vs. ADSC-Exo+E | -0.4425 | Yes | **** | <0.0001 | B-C |  | |  |  |  |  |
| Fig4-I-LPO | | | | | | | |  |  |  |  |
| KF vs. ADSC-Exo | 0.5102 | Yes | **** | <0.0001 | A-B |  | |  |  |  |  |
| KF vs. ADSC-Exo+E | -0.2534 | Yes | **** | <0.0001 | A-C |  | |  |  |  |  |
| ADSC-Exo vs. ADSC-Exo+E | -0.7636 | Yes | **** | <0.0001 | B-C |  | |  |  |  |  |
| Fig4-J-GSH | | | | | | | |  |  |  |  |
| KF vs. ADSC-Exo | -1.040 | Yes | *** | 0.0002 | A-B |  | |  |  |  |  |
| KF vs. ADSC-Exo+E | 0.4320 | No | ns | 0.0550 | A-C |  | |  |  |  |  |
| ADSC-Exo vs. ADSC-Exo+E | 1.472 | Yes | **** | <0.0001 | B-C |  | |  |  |  |  |
| Fig5-G-α-SMA | | | | | | | |  |  |  |  |
| Bonferroni's multiple comparisons test | Mean Diff. | 95.00% CI of diff. | Below threshold? | Summary | Adjusted P Value |  | |  |  |  |  |
| Control vs. ADSC-Exo | 9.445 | 1.332 to 17.56 | Yes | * | 0.0371 | A-B | |  |  |  |  |
| Control vs. ADSC-Exo+E | -4.293 | -16.96 to 8.373 | No | ns | 0.3663 | A-C | |  |  |  |  |
| ADSC-Exo vs. ADSC-Exo+E | -13.74 | -23.12 to -4.354 | Yes | * | 0.0236 | B-C | |  |  |  |  |
| Fig5-H-COL1A1 | | | | | | | |  |  |  |  |
| Control vs. ADSC-Exo | 10.43 | 2.318 to 18.54 | Yes | * | 0.0178 | A-B | |  |  |  |  |
| Control vs. ADSC-Exo+E | -3.342 | -11.45 to 4.771 | No | ns | 0.4630 | A-C | |  |  |  |  |
| ADSC-Exo vs. ADSC-Exo+E | -13.77 | -21.89 to -5.660 | Yes | ** | 0.0048 | B-C | |  |  |  |  |
| Fig5-I-COL3A1 | | | | | | | |  |  |  |  |
| Control vs. ADSC-Exo | -20.13 | -27.15 to -13.10 | Yes | *** | 0.0003 | A-B | |  |  |  |  |
| Control vs. ADSC-Exo+E | -8.209 | -15.24 to -1.181 | Yes | * | 0.0269 | A-C | |  |  |  |  |
| ADSC-Exo vs. ADSC-Exo+E | 11.92 | 4.889 to 18.94 | Yes | ** | 0.0048 | B-C | |  |  |  |  |
| Fig5-J-GPX4 | | | | | | | |  |  |  |  |
| Control vs. ADSC-Exo | -19.10 | -29.00 to -9.194 | Yes | ** | 0.0025 | A-B | |  |  |  |  |
| Control vs. ADSC-Exo+E | -1.055 | -10.96 to 8.848 | No | ns | 0.9433 | A-C | |  |  |  |  |
| ADSC-Exo vs. ADSC-Exo+E | 18.04 | 8.139 to 27.95 | Yes | ** | 0.0034 | B-C | |  |  |  |  |
| Fig6-B-α-SMA | Mean Diff. | Below threshold? | Summary | Adjusted P Value |  |  | |  |  |  |  |
| Control vs. ADSC-Exo | 0.8336 | Yes | **** | <0.0001 | A-B |  | |  |  |  |  |
| Control vs. ADSC-Exo+E | 0.4459 | Yes | **** | <0.0001 | A-C |  | |  |  |  |  |
| ADSC-Exo vs. ADSC-Exo+E | -0.3877 | Yes | **** | <0.0001 | B-C |  | |  |  |  |  |
| Fig6-C-COL1A1 | | | | | | | |  |  |  |  |
| Control vs. ADSC-Exo | 0.6998 | Yes | * |  | A-B |  | |  |  |  |  |
| Control vs. ADSC-Exo+E | -0.2136 | No | ns |  | A-C |  | |  |  |  |  |
| ADSC-Exo vs. ADSC-Exo+E | -0.9133 | Yes | * |  | B-C |  | |  |  |  |  |
| Fig6-D-COL3A1 | Mean Diff. | 95.00% CI of diff. | Below threshold? | Summary | Adjusted P Value |  | |  |  |  |  |
| Control vs. ADSC-Exo | -0.5741 | -1.053 to -0.09509 | Yes | * | 0.0197 | A-B | |  |  |  |  |
| Control vs. ADSC-Exo+E | 0.06577 | -0.4133 to 0.5448 | No | ns | >0.9999 | A-C | |  |  |  |  |
| ADSC-Exo vs. ADSC-Exo+E | 0.6399 | 0.1609 to 1.119 | Yes | * | 0.0106 | B-C | |  |  |  |  |
| Fig6-E-COL1A1/ COL3A1 | Mean Diff. | Below threshold? | Summary | Adjusted P Value |  |  | |  |  |  |  |
| Control vs. ADSC-Exo | 0.7998 | Yes | * | 0.0499 | A-B |  | |  |  |  |  |
| Control vs. ADSC-Exo+E | -0.3117 | No | ns | 0.3237 | A-C |  | |  |  |  |  |
| ADSC-Exo vs. ADSC-Exo+E | -1.111 | Yes | * | 0.0142 | B-C |  | |  |  |  |  |
| Fig6-G-GPX4 | Mean Diff. | 95.00% CI of diff. | Below threshold? | Summary | Adjusted P Value |  | |  |  |  |  |
| Control vs. ADSC-Exo | -5.170 | -8.748 to -1.592 | Yes | ** | 0.0075 | A-B | |  |  |  |  |
| Control vs. ADSC-Exo+E | -1.347 | -4.925 to 2.231 | No | ns | 0.5655 | A-C | |  |  |  |  |
| ADSC-Exo vs. ADSC-Exo+E | 3.823 | 0.2446 to 7.401 | Yes | * | 0.0371 | B-C | |  |  |  |  |
| Fig6-H-MDA | Mean Diff. | Below threshold? | Summary | Adjusted P Value |  |  | |  |  |  |  |
| Control vs. ADSC-Exo | 1.541 | Yes | * | 0.0399 | A-B |  | |  |  |  |  |
| Control vs. ADSC-Exo+E | -0.7596 | No | ns | 0.1731 | A-C |  | |  |  |  |  |
| ADSC-Exo vs. ADSC-Exo+E | -2.301 | Yes | * | 0.0101 | B-C |  | |  |  |  |  |
| Fig6-I-LPO | Mean Diff. | 95.00% CI of diff. | Below threshold? | Summary | Adjusted P Value |  | |  |  |  |  |
| Control vs. ADSC-Exo | 2.494 | 2.245 to 2.742 | Yes | **** | <0.0001 | A-B | |  |  |  |  |
| Control vs. ADSC-Exo+E | 1.638 | 1.389 to 1.887 | Yes | **** | <0.0001 | A-C | |  |  |  |  |
| ADSC-Exo vs. ADSC-Exo+E | -0.8553 | -1.104 to -0.6064 | Yes | *** | 0.0001 | B-C | |  |  |  |  |
| Fig6-J-GSH |  |  |  |  |  |  | |  |  |  |  |
| Control vs. ADSC-Exo | -0.6724 | -1.190 to -0.1550 | Yes | * | 0.0170 | A-B | |  |  |  |  |
| Control vs. ADSC-Exo+E | -0.09750 | -0.6150 to 0.4200 | No | ns | 0.8364 | A-C | |  |  |  |  |
| ADSC-Exo vs. ADSC-Exo+E | 0.5749 | 0.05746 to 1.092 | Yes | * | 0.0331 | B-C | |  |  |  |  |
| Fig7-B-α-SMA | | | | | | | |  |  |  |  |
| control vs. ADSC-Exo | 0.5779 | 0.2954 to 0.8605 | Yes | *** | 0.0006 | A-B | |  |  |  |  |
| control vs. sinc+ADSC-Exo | 0.4514 | 0.1689 to 0.7340 | Yes | ** | 0.0032 | A-C | |  |  |  |  |
| control vs. sis+ADSC-Exo | 0.1634 | -0.1192 to 0.4459 | No | ns | 0.4746 | A-D | |  |  |  |  |
| ADSC-Exo vs. sinc+ADSC-Exo | -0.1265 | -0.4091 to 0.1561 | No | ns | 0.9481 | B-C | |  |  |  |  |
| ADSC-Exo vs. sis+ADSC-Exo | -0.4145 | -0.6971 to -0.1320 | Yes | ** | 0.0056 | B-D | |  |  |  |  |
| sinc+ADSC-Exo vs. sis+ADSC-Exo | -0.2881 | -0.5706 to -0.005491 | Yes | * | 0.0453 | C-D | |  |  |  |  |
|  |  |  |  |  |  |  | |  |  |  |  |
| Fig7-C-COL1A1 |  |  |  |  |  |  | |  |  |  |  |
| control vs. ADSC-Exo | 0.3212 | -0.06812 to 0.7106 | No | ns | 0.1207 | A-B | |  |  |  |  |
| control vs. sinc+ADSC-Exo | 0.2976 | -0.09176 to 0.6870 | No | ns | 0.1637 | A-C | |  |  |  |  |
| control vs. sis+ADSC-Exo | -0.2212 | -0.6106 to 0.1681 | No | ns | 0.4113 | A-D | |  |  |  |  |
| ADSC-Exo vs. sinc+ADSC-Exo | -0.02364 | -0.4130 to 0.3657 | No | ns | >0.9999 | B-C | |  |  |  |  |
| ADSC-Exo vs. sis+ADSC-Exo | -0.5425 | -0.9318 to -0.1531 | Yes | ** | 0.0078 | B-D | |  |  |  |  |
| sinc+ADSC-Exo vs. sis+ADSC-Exo | -0.5188 | -0.9082 to -0.1295 | Yes | * | 0.0103 | C-D | |  |  |  |  |
| Fig7-D-COL3A1 | Mean Diff. | Below threshold? | Summary | Adjusted P Value |  |  | |  |  |  |  |
| control vs. ADSC-Exo | -0.4176 | Yes | * | 0.0169 | A-B |  | |  |  |  |  |
| control vs. sinc+ADSC-Exo | -0.1920 | No | ns | 0.1268 | A-C |  | |  |  |  |  |
| control vs. sis+ADSC-Exo | 0.3419 | Yes | * | 0.0357 | A-D |  | |  |  |  |  |
| ADSC-Exo vs. sinc+ADSC-Exo | 0.2256 | No | ns | 0.1268 | B-C |  | |  |  |  |  |
| ADSC-Exo vs. sis+ADSC-Exo | 0.7595 | Yes | *** | 0.0006 | B-D |  | |  |  |  |  |
| sinc+ADSC-Exo vs. sis+ADSC-Exo | 0.5339 | Yes | ** | 0.0050 | C-D |  | |  |  |  |  |
|  |  |  |  |  |  |  | |  |  |  |  |
| Fig7-E-COL1A1/ COL3A1 |  |  |  |  |  |  | |  |  |  |  |
| control vs. ADSC-Exo | 0.5672 | Yes | ** | 0.0019 | A-B |  | |  |  |  |  |
| control vs. sinc+ADSC-Exo | 0.5381 | Yes | ** | 0.0020 | A-C |  | |  |  |  |  |
| control vs. sis+ADSC-Exo | -0.4465 | Yes | ** | 0.0042 | A-D |  | |  |  |  |  |
| ADSC-Exo vs. sinc+ADSC-Exo | -0.02903 | No | ns | 0.7791 | B-C |  | |  |  |  |  |
| ADSC-Exo vs. sis+ADSC-Exo | -1.014 | Yes | **** | <0.0001 | B-D |  | |  |  |  |  |
| sinc+ADSC-Exo vs. sis+ADSC-Exo | -0.9847 | Yes | **** | <0.0001 | C-D |  | |  |  |  |  |
| Fig7-F-GPX4 |  |  |  |  |  |  | |  |  |  |  |
| control vs. ADSC-Exo | -0.6480 | Yes | * | 0.0405 | A-B |  | |  |  |  |  |
| control vs. sinc+ADSC-Exo | -0.8505 | Yes | * | 0.0125 | A-C |  | |  |  |  |  |
| control vs. sis+ADSC-Exo | 0.1615 | No | ns | 0.5596 | A-D |  | |  |  |  |  |
| ADSC-Exo vs. sinc+ADSC-Exo | -0.2025 | No | ns | 0.4674 | B-C |  | |  |  |  |  |
| ADSC-Exo vs. sis+ADSC-Exo | 0.8095 | Yes | * | 0.0158 | B-D |  | |  |  |  |  |
| sinc+ADSC-Exo vs. sis+ADSC-Exo | 1.012 | Yes | * | 0.0051 | C-D |  | |  |  |  |  |
| Fig7-G-SLC7A11 | Mean Diff. | 95.00% CI of diff. | Below threshold? | Summary | Individual P Value |  | |  |  |  |  |
| control vs. ADSC-Exo | -0.6872 | -1.330 to -0.04419 | Yes | * | 0.0398 | A-B | |  |  |  |  |
| control vs. sinc+ADSC-Exo | -0.6900 | -1.333 to -0.04696 | Yes | * | 0.0393 | A-C | |  |  |  |  |
| control vs. sis+ADSC-Exo | 0.5901 | -0.05290 to 1.233 | No | ns | 0.0658 | A-D | |  |  |  |  |
| ADSC-Exo vs. sinc+ADSC-Exo | -0.002775 | -0.6458 to 0.6402 | No | ns | 0.9919 | B-C | |  |  |  |  |
| ADSC-Exo vs. sis+ADSC-Exo | 1.277 | 0.6343 to 1.920 | Yes | ** | 0.0028 | B-D | |  |  |  |  |
| sinc+ADSC-Exo vs. sis+ADSC-Exo | 1.280 | 0.6371 to 1.923 | Yes | ** | 0.0028 | C-D | |  |  |  |  |
